# Filled prescriptions of age-related contraindicated drugs in children: a one-year nationwide cohort study in the Netherlands

**DOI:** 10.1007/s11096-018-0717-6

**Published:** 2018-08-23

**Authors:** K. Cheung, M. Teichert, H. A. Moll, B. H. Stricker, L. E. Visser

**Affiliations:** 1 Department of Epidemiology, Erasmus Medical Center, PO Box 2040, 3000 CA Rotterdam, The Netherlands; 2 Health and Youth Care Inspectorate, Utrecht, The Netherlands; 3 Department of Clinical Pharmacy and Toxicology, Leiden University Medical Center, Leiden, The Netherlands; 4 Department of Pediatrics, Erasmus MC-Sophia Children’s Hospital, Rotterdam, The Netherlands; 5 Haga Teaching Hospital, Rotterdam, The Netherlands

**Keywords:** Age, Children, Contraindication, Dispensing, Netherlands, Paediatrics

## Abstract

*Background* Children are still prescribed age contraindicated drugs, but information about the number and type of these drugs dispensed for children in the Netherlands is limited. *Objective* To determine the incidence and prevalence of contraindicated drugs that were dispensed for the use by children. *Setting* The study was conducted in the Netherlands with routinely collected data from 95% of all community pharmacies. *Method* We performed a one-year nationwide observational study where all patients aged 17 years or younger who have received at least one prescription in 2016 were included. Contraindicated drugs were selected, according to the 5th level of ATC code, using different information sources. *Main outcome measure* The proportion of (newly) contraindicated drugs that were dispensed to children. *Results* In total, 3.9% of all children received at least one drug that was contraindicated for their age. The highest percentage of contraindicated drugs that was dispensed, was observed in patients aged 1–2 years and 13–17 years (7.0 and 5.7%, respectively) and the percentage of contraindicated drugs that were dispensed was higher in female than in male patients (4.3 and 3.6%, respectively; *p* value < 0.001). *Conclusion* The results of this study show that a substantial percentage of children received a drug that was conta-indicated for their age, and it happes more in female than in male patients. Furthermore, the information about this type of contraindications is limited and inconsistent.

## Impact of findings

- Information about contraindications or warnings should be improved to support prescribers and other healthcare professionals when prescribing contraindicated drugs which are deemed necessary.
- It is not clear why contraindicated drugs are more often prescribed for female than male children. Further research in this field should be carried out to address these gender differences and to improve our knowledge about the safety of drugs  in thse patient groups.

## Introduction

Many drugs are prescribed off-label because they are not approved for use in children [1, 2]. GPs and other prescribers make decisions based on the available evidence, which can be very limited. Drugs used for treating children are often insufficiently documented with regard to dosing, efficacy, and safety in this patient group. When off-label use is associated with a safety hazard or a risk of serious adverse drug reactions, it is described as a contraindication, which means that there is sufficient evidence that the drug is or might be harmful and use of these drugs is not advised. [3]. In some circumstances there is insufficient information available about the use of drugs in children. In this case, it is acceptable to prescribe these drugs if the benefits outweigh the risks (warning). Contraindications are always described in the labels of the products concerned and should be strictly followed to ensure safe use of these drugs in a specific population [4].

One of the recent examples is the label revision for products containing codeine and tramadol which states that these drugs are contraindicated for the treatment of pain in children younger than age 12. Adverse event reports showed that the use of codeine was associated with respiratory depression and death, with the majority of cases involving children under the age of 12 [5]. This contraindication shows that it is important for healthcare professionals to know that prescribing these drugs to children from this particular age group should be avoided and alternatives should be used instead. However, in some cases, contraindicated drugs are still prescribed when there is a great burden of disease and no alternatives are available [6]. In other cases it is also possible that prescribers were not aware of the contraindications in the drug labelling [6].

Numerous studies have described the contraindication of medication use in relation to specific diseases or patient characteristics, but the focus on age and gender in children in these studies is very limited [7–9]. Moreover, these studies are often based on prescription data and it is not always clear if these drugs were actually dispensed.

## Aim of the study

To determine the incidence and prevalence of contraindicated drugs dispensed to children, with routinely collected data from community pharmacies in the Netherlands.

## Ethics approval

Informed consent was not required, since this was an observational, non-interventional cohort study.

## Method

### Setting

Data were obtained from the Dutch Foundation for Pharmaceutical Statistics (Stichting Farmaceutische Kengetallen). This database contains dispensing data from 95% of all community pharmacies in the Netherlands since 1990 [10]. In the Netherlands, the community pharmacies dispense the vast majority of all out-patient prescriptions. For each dispensing, the following information is available: a unique anonymised patient code, gender and year of birth, product name, active substance according to the Anatomic Therapeutical Chemical code (ATC code) [11], dispensing date, total number of drug units per prescription, prescribed daily number of units, dosage, regimen, type of prescriber (GP, specialist or other healthcare professional) and the first two digits of the postal code indicating the region.

### Cohort definition

All patients aged 17 years or younger who have received at least one prescription during the study period between 1 January 2016 and 31 December 2016 were included. Patients with an unknown gender were excluded.

### Measures

Drugs that are contraindicated in children were selected according to the 5^th^ level ATC code of the World Health Organisation [11]. The prescription codes (PRK code) were used if products had to be selected on substance level. The primary reference source that was used to determine the contraindication status in children was the Farmacotherapeutisch Kompas 2016 (a national formulary provided by the National Health Care Institute) because it contains information of all drugs and most details about the topic of interest [12]. The descriptions in the Farmacotherapeutisch Kompas are written based on the information in the summary of product characteristcs (SmPC), which is considered as a legal document that was approved by the Medicines Evaluation Board or the European Medicines Agency. The alternative source of information was the SmPC of the products concerned, which was used in case of conflicting information from different sources. Furthermore, the Kinderformularium (national formulary for children) was used when the information about the contraindication was not clearly described [13]. The following drugs that are contraindicated in children below 6 months of age were excluded from the analysis as only the year of birth was available: atazanavir, cotrimoxazol, fluticasone, hydrocortison (systemic), methylprednisolone, nandrolone, sodium polystyrene sulfonate, pethidine, prilocaine, somatropin, sulfadiazine, tocofersolan and vitamine B complex (parenteral).

### Analysis

For all patients, we determined the number of drugs dispensed between 1 January 2016 and 31 December 2016. In our study, a person was identified as user of a contraindicated drug when having at least one contraindicated drug dispensed during the study period. In the analyses, we have calculated the cumulative 1-year incidence as the proportion of the total cohort to which a contraindicated drug was dispensed in the year 2016 for the first time since birth. In addition, the cumulative 1-year prevalence was determined by the proportion of children with a contraindicated drug dispensed in the study period, including those who had started before 1 January 2016. This was also stratified according to age, which was classified into five groups: < 1 year, 1–2 years, 3–6 years, 7–12 years and 13–17 years. In addition, we investigated the gender differences and we calculated the number of contraindicated drugs that were dispensed per region and per type of prescriber. The analyses included almost the entire Dutch population and therefore the calculation of confidence intervals for the incidence and prevalence were not deemed necessary. We also investigated the information available in the Farmacotherapeutisch Kompas, SmPC, Kinderformularium about the reasons for a drug to be contraindicated, which are classified into the active ingredient, high/unknown dosage, formulation, lack of information about efficacy or safety and the presence of excipients. Finally, we have calculated the number of patients, the number of drugs that were dispensed and mean age for the most commonly dispensed contraindicated drugs.

## Results

Patients with an unknown gender (n = 122,144) were excluded from the analysis. The remaining 1,527,021 patients received at least one drug in 2016 where 3.9% of these patients received a drug that was contraindicated for age (Table 1). The cumulative prevalence was significantly higher in females compared to males (4.3% and 3.6%, respectively; *p* value < 0.001) and highest in patients aged 1–2 years (7.0%). The majority of patients received only one type of drug that was contraindicated for age in the study period (1 drug: 58,597 (6.0%); 2 or more: 1,496 (0.3%)).

**Table 1:** Children aged 0–17 years who were prescribed contraindicated drugs in 2016

	Number of children who received drugs	Number of children who received a contraindicated drug (%)	Number of dispensed drugs	Number of dispensed contraindicated drugs (%)
Overall	1,527,021	60,093 (3.9)	4,454,048	105,106 (2.3)
*Gender*				
Male	769,903	27,604 (3.6)	2,370,735	48,481 (2.0)
Female	757,118	32,489 (4.3)	2,083,313	56,625 (2.7)
*Age*				
< 1 year	59,163	2,289 (3.9)	129,022	2,901 (2.2)
1–2 years	214,506	15,114 (7.0)	277,841	21,295 (7.6)
3–6 years	336,202	3,093 (0.9)	762,978	5,198 (0.7)
7–12 years	429,485	11,731 (2.7)	1,253,424	20,456 (1.6)
13–17 years	487,665	27,866 (5.7)	2,030,783	55,256 (2.7)
*Number of different drugs* ^a^				
1 drug	972,451	58,597 (6.0)	–	–
2 or more	554,570	1,496 (0.3)	–	–

^a^The number of different drugs was determined based on the ATC7-codes

The number of new users of a contraindicated drug in 2016 was 43,206 (2.8%), which was significantly higher in females (3.1%) than in males (2.6%) (*p* value < 0.001). The cumulative incidence was highest in the age categories 1–2 years (5.5%) and 13–17 years (4.2%), followed by the age categories 0–1 (2.3%), 7–12 years (1.8%) and 3–6 years (0.6%). The postal code information available for each patient indicated that the number of patients who received a contraindicated drug in the Netherlands is fairly equally distributed (North 4.0%; East 3.9%; West 3.9% and South 4.0%). The majority of patients received the contraindicated drugs from their GP (n = 1,256,213), followed by medical specialists (n = 314,836) and other healthcare professionals (n = 206,563) but the contraindicated drugs were nevertheless relatively more often prescribed by medical specialists than by GPs (GP 3.4%, specialist 4.2% and other 3.6%). The most common drugs that were relatively more often prescribed by specialist than GPs or other healthcare professionals are hydrocortisone/neomycin/polymyxin B (specialist: 1.26%, GP: 0.48% and other healthcare professionals: 0.37%), atropine (specialist: 0.26%; GP:0.003% and other healthcare professionals: 0.03%) and dimetindene (specialist: 0.23%; GP:0.06% and other: 0.08%).

Overall, the number of patients who received a contraindicated drug was significantly higher in females than in males (4.3 and 3.6%, respectively; *p* value < 0.001) (Table 2). Higher percentages were observed for female patients in the higher age groups (7–12 years: 3.0%, 13–17 years: 6.5%) compared with male patients (7–12 years: 2.5%, 13–17 years: 4.6%). For male patients, higher numbers were observed in the lower age groups (< 1 year: 4.1%, 1–2 years: 7.7%) than in female patients (< 1 year: 3.6%, 1–2 years: 6.2%). Cholecalciferol (females: 2.5% and males 1.5%) and metoclopramide (females: 0.5% and males: 0.3%) were relatively more often prescribed to females than males. Hydrocortisone/neomycine/polymyxine B (males: 0.8% and females: 0.5%) and dimetidene (males: 0.13% and females: 0.08%) were relatively more often prescribed to male patients.

**Table 2:** Children who were prescribed contraindicated drugs per age group, stratified by gender

Age	Male		Female	
	Total	Contraindicated (%)	Total	Contraindicated (%)
All ages	769,903	27,604 (3.6)	757,118	32,489 (4.3)
<1 year	33,201	1357 (4.1)	25,962	932 (3.6)
1–2 years	116,332	9007 (7.7)	98,174	6107 (6.2)
3–6 years	177,066	1668 (0.9)	159,136	1425 (0.9)
7–12 years	232,991	5812 (2.5)	196,494	5919 (3.0)
13–17 years	210,313	9760 (4.6)	277,352	18,106 (6.5)

One of the most common contraindications is due to the active pharmaceutical ingredient, which is seen in 49 types of drugs and in 25,875 patients (Table 3). Drugs that are contraindicated due to the formulation (e.g. difficulty swallowing) was only observed in one type of drug and in 180 patients.

**Table 3:** Different type of reasons for age-related contraindications

Reason for contraindication	Number of different types of drugs with a contraindication for age (n = 63)* n (%)	Number of patients receiving a contraindicated drug (n = 60,093) n (%)
Active ingredient	49 (77.8)	25,875 (43.1)
High dosage or unknown dosage	7 (11.1)	32,323 (53.8)
Formulation	1 (1.6)	180 (0.3)
Lack of information about efficacy or safety	3 (4.8)	1675 (2.8)
Presence of excipients	3 (4.8)	40 (0.1)

*Only contraindicated drugs that were dispensed to children by community pharmacies in the Netherlands in 2016 were included

*n* number

**Fig. 1:**
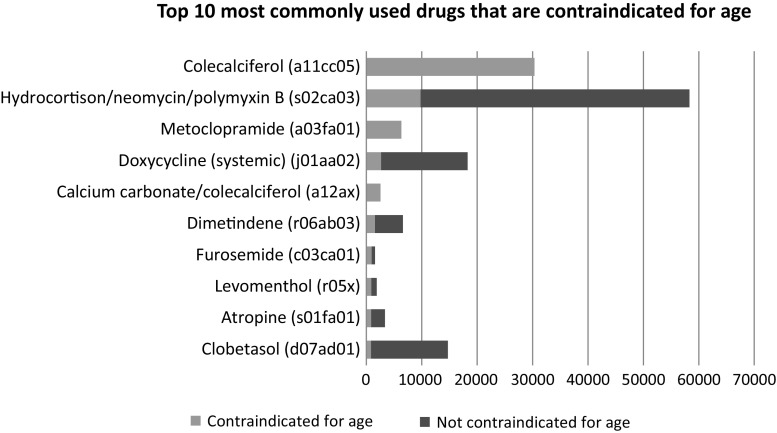
The 10 most commonly prescribed drugs with a contraindication for age: users within the contraindication age limit versus users outside the contraindication age limit in absolute numbers

Figure 1 shows that cholecalciferol is one of the most frequently dispensed drugs that is contraindicated for age. The combination of hydrocortisone/neomycin/polymyxin b, doxycycline, dimetindene, atropine and clobetasol were also in the top ten of the most commonly dispensed contraindicated drugs, but these drugs were relatively less dispensed to children with an age that is within the contraindication age limit. The other 15 most commonly dispensed drugs were: fludrocortisone/neomycine/polymyxin B/lidocaine, lidocaine, promethazine, etoricoxib, meloxicam, levodopa/benserazide, temazepam, zolpidem, insulin, zopiclone, rhubarb extract/salicylic acid, loperamide, scopolamine, norfloxacin (systemic) and sodium phosphate (rectal) (results not shown due to low numbers).

Table 4 shows more detailed information from the most commonly dispensed drugs with a contraindication for age. For a number of drugs the actual number of contraindicated drugs dispensed is lower than the group that was selected based on the ATC codes. Cholecalciferol was dispensed to 30,301 children, where 15,988 children (53%) received the oral suspension which is contraindicated because of its high dose of vitamin D. Metoclopramide was dispensed to 6253 patients where 110 patients (1.8%) were below the age of 1. The metoclopramide suppositories which are contraindicated in children and adolescents less than 18 years, were dispensed to 3559 children (57% of the total number). Doxycycline was dispensed to 2,670 children with an age lower than 12 years, of whom 305 were less than 8 years (11.4%). Furthermore, 268 patients received the drug with the ATC code N04BA02 which not only includes levodopa/benserazide, but also levodopa/carbidopa. The contraindicated drug levodopa/benserazide was dispensed to 93 patients (35%). Other commonly dispensed drugs that are contraindicated in children below the age of 2 years are dimetindene, levomenthol, promethazine, loperamide, fludrocortisone/neomycin/polymyxin B and lidocaine. Temazepam and zoplicon are contraindicated for age because the effectiveness and safety of use in children have not been studied yet.

**Table 4:** Additional information on the most commonly prescribed contraindicated drugs

Name of drug (ATC code)	Children^a^	Dispensings^b^	Mean age (SD)	Potential risk, reason for contraindication
Cholecalciferol (A11CC05)^c^	30,301	60,034	13.3 (4.0)	The oral suspension of cholecalciferol and the combination with calcium carbonate is contraindicated in children aged 17 years and below because it contains a high dose of Vitamin D
Calcium carbonate/cholecalciferol (A12AX)	2518	6434	12.7 (3.9)	
Metoclopramide (A03FA01)	6253	7056	12.0 (4.8)	Contraindicated in children below the age of 1. Metoclopramide suppositories are contraindicated in children aged 17 and below because of the high risk of extrapyramidal disorders
Doxycycline (systemic) (J01AA02)	2670	2904	10.4 (1.8)	Contraindicated in children below the age of 8 (infections) and 12 years (facial rosacea), because of the risk of tooth staining
Dimetindene (R06AB03)	1560	2106	0.8 (0.4)	Contraindicated in children below the age of 1, because the sedative effect can be associated with episodes of sleep apnoea
Levomenthol (R05X)	904	936	0.9 (0.7)	Contraindicated in children below the age of 2, because substances containing menthol have been reported to cause instant collapse or laryngospasm
Fludrocortisone/neomycin/polymyxine B/lidocaine combination (S02CA07)	800	891	1.4 (0.6)	Contraindicated in children below the age of 2 because of the possibility of increased absorption of neomycine and the kidney function may not be fully developed. There is also a potential risk of nephrotoxicity and ototoxicity due to neomycin
Promethazine (R06AD02)	643	696	1.9 (0.4)	Contraindicated in children below the age of 2, because promethazine may lead to severe respiratory depression and apnea. A potential association of promethazine use with the sudden infant death syndrome has also been reported
Levodopa/Benserazide combination (N04BA02)	268	496	11.4 (4.8)	Contraindicated in children and adolescents aged 24 years and below. Animal studies have suggested that benserazide may cause skeletal abnormalities if administered before skeletal development is complete
Temazepam (N05CD07)^c^	226	352	7.0 (3.4)	Contraindicated in children aged 11 years and below because the safety and effectiveness in children have not been established
Zoplicon (N05CF01)	171	283	14.4 (3.9)	
Loperamide (A07DA03)	112	129	1.3 (0.8)	Contraindicated in children below the age of 2 because use of loperamide has been associated with fatal episodes of paralytic ileus

^a^The number of patients are counted based on the ATC codes. The numbers of patients who have received the medication within the age limit for contraindication are shown. Patients outside the age limit were excluded

^b^The number of drugs dispensed are counted based on the ATC codes. The number of drugs dispensed are shown of drugs that are contraindicated for age

^c^Cholecalciferol and the combination with calcium carbonate have been compiled on one line. Temazepam and Zoplicon have been compiled on one line

*ATC* anatomical therapeutic chemical, *SD* standard deviation

## Discussion

It is not always clear why a drug is contraindicated and how many of these drugs are prescribed to children despite their contraindication for age. In this nationwide study, we have calculated the incidence and prevalence of drugs with a contraindication for the age groups to which they were nevertheless dispensed. In our study we observed that 3.9% of all children received at least one drug that was contraindicated for age, which are mainly newly prescribed drugs (2.8%). These results are almost similar to the results observed in the study by Bensouda-Grimaldi et al. (3.2%) [6]. Our results also showed that male patients received more overall prescriptions than female patients, but contraindicated drugs were relatively more often dispensed to female patients. The difference in drug use may partly be explained by gender differences such as the variation in disease prevalence or severity, but the differences in health care may also explain this observation [14–17]. Further research is needed to address the gender differences in prescribing drugs to children.

Alternatives are available for many contraindicated drugs, but in some circumstances the contraindicated drug is needed. Prescribing an on-label drug does not always have to be accurate, whereas prescribing a contraindicated drug does not have to be considered bad practice either. One of the examples is the oral suspension cholecalciferol which is contraindicated because of the high dose of vitamin D. These drugs are necessary for children with an impaired vitamin D absorption such as cystic fibrosis or serious vitamin D deficiency in rachitis. However, it is not clear if they were all prescribed for these type of indications, as we observed a large number of patients who received the high dose of Vitamin D in our study. The increase of vitamin D use since the global recognition of vitamin D deficiency in the general population has also led to an increase in vitamin D intoxications, which were partly due to inappropriate prescribing [18]. This may also be explained by the differences between information sources that was observed as the oral suspension with a high dose of vitamin D was only mentioned in the Farmacotherapeutisch Kompas, but not mentioned or described in the Kinderformulary. Specialists, GPs or other healthcare professionals may use different information sources as we also observed that not only specialist were more likely to prescribe contraindicated drugs, but also the type of drugs between specialists and GPs together with other healthcare professionals were slightly different.

Metoclopramide is still prescribed for chronic use for the indication symptomatic gastroesophageal reflux as the substantial need is not met by other medications [19]. However, the contraindication of metoclopramide suppositories in higher age groups is not very clear as it is described as a contraindication, but was also described as a warning according to the same information source [20]. Another commonly prescribed drug that is contraindicated in children is doxycycline, which should be avoided because of its known adverse effect; tooth staining [21]. It is possible that doxycycline is still prescribed despite the label warning as it is considered as one of the most effective treatments for tick borne diseases or because recent studies showed no visible dental staining in children [22–24].

### Strengths and limitations

One of the strengths of our study is that we used a population-based dispensing database which registers drugs that were dispensed in more than 95% of all community pharmacies in the Netherlands. Pharmacists play an essential role in drug safety and it is likely that the number of contraindicated drugs is higher in prescription data than in our study as we have used dispensing data. Detailed information was available on all drugs that were dispensed to children. The relatively long history available also enabled us to study any drugs that were dispensed since birth to determine the 1-year cumulative incidence and prevalence.

Our study also has several limitations. Our data do not include the drugs that were dispensed by hospital pharmacies where the numbers of contraindicated drugs that were dispensed may be higher. No information about the month of birth was available. Therefore, we only included drugs in our analysis where the contraindication for age was based on the number of years and not months. Also, no information was available with regard to the indication of the drugs prescribed.

## Conclusion

The results of this study showed that a substantial percentage of children received a dispensed drug which is contraindicated for that age. This is more common in females than in males. Gender differences in disease prevalence or healthcare may explain this observation. We also observed that the information about the contraindication (contraindication or warning and the reason) is limited and not consistent between the different information sources. Since prescribing an on-label drug can be inaccurate, as much as prescribing an contraindication drug can be good practice, it is important that all prescribers and healthcare professionals are informed sufficiently about the contraindications. Further research is needed to investigate the reasons for prescribing particular drugs despite their contraindication for age, and the gender differences that were found in this study.
